# Supplementary material for: Pyruvate Dehydrogenase Kinase 1 inhibition mediated oxidative phosphorylation enhancement in cartilage promotes osteoarthritis progression
Source: BMC Musculoskelet Disord. 2023 Jul 20;24:597. doi: 10.1186/s12891-023-06585-6 (PMC10357736; doi:10.1186/s12891-023-06585-6)
Supplement: Supplementary file 1 — Additional file 1. [file 12891_2023_6585_MOESM1_ESM.zip › MF.Gsea.1653627316242/GOMF_DNA_BINDING_TRANSCRIPTION_REPRESSOR_ACTIVITY.html]

Details for gene set GOMF\_DNA\_BINDING\_TRANSCRIPTION\_REPRESSOR\_ACTIVITY[GSEA]

|  || Dataset | 55457\_collapsed\_to\_symbols.cls.cls #OA\_versus\_normal.cls.cls #OA\_versus\_normal\_repos |
| Phenotype | cls.cls#OA\_versus\_normal\_repos |
| Upregulated in class | normal |
| GeneSet | GOMF\_DNA\_BINDING\_TRANSCRIPTION\_REPRESSOR\_ACTIVITY |
| Enrichment Score (ES) | -0.36201888 |
| Normalized Enrichment Score (NES) | -1.7625206 |
| Nominal p-value | 0.0 |
| FDR q-value | 0.07692162 |
| FWER p-Value | 0.698 |
Table: GSEA Results Summary

  

Fig 1: Enrichment plot: GOMF\_DNA\_BINDING\_TRANSCRIPTION\_REPRESSOR\_ACTIVITY      
 Profile of the Running ES Score & Positions of GeneSet Members on the Rank Ordered List

  

| SYMBOL | TITLE | RANK IN GENE LIST | RANK METRIC SCORE | RUNNING ES | CORE ENRICHMENT || 1 | ZNF529 | zinc finger protein 529 [Source:HGNC Symbol;Acc:HGNC:29328] | 75 | 0.954 | 0.0064 | No |
| 2 | THAP1 | THAP domain containing 1 [Source:HGNC Symbol;Acc:HGNC:20856] | 153 | 0.856 | 0.0114 | No |
| 3 | MSX2 | msh homeobox 2 [Source:HGNC Symbol;Acc:HGNC:7392] | 169 | 0.839 | 0.0210 | No |
| 4 | HHEX | hematopoietically expressed homeobox [Source:HGNC Symbol;Acc:HGNC:4901] | 270 | 0.774 | 0.0231 | No |
| 5 | PAX6 | paired box 6 [Source:HGNC Symbol;Acc:HGNC:8620] | 304 | 0.746 | 0.0302 | No |
| 6 | BHLHE41 | basic helix-loop-helix family member e41 [Source:HGNC Symbol;Acc:HGNC:16617] | 417 | 0.700 | 0.0304 | No |
| 7 | SOX30 | SRY-box transcription factor 30 [Source:HGNC Symbol;Acc:HGNC:30635] | 503 | 0.658 | 0.0322 | No |
| 8 | TCFL5 | transcription factor like 5 [Source:HGNC Symbol;Acc:HGNC:11646] | 599 | 0.623 | 0.0327 | No |
| 9 | HOXB8 | homeobox B8 [Source:HGNC Symbol;Acc:HGNC:5119] | 652 | 0.605 | 0.0364 | No |
| 10 | HESX1 | HESX homeobox 1 [Source:HGNC Symbol;Acc:HGNC:4877] | 656 | 0.604 | 0.0440 | No |
| 11 | NR3C1 | nuclear receptor subfamily 3 group C member 1 [Source:HGNC Symbol;Acc:HGNC:7978] | 674 | 0.599 | 0.0504 | No |
| 12 | ZNF557 | zinc finger protein 557 [Source:HGNC Symbol;Acc:HGNC:28632] | 846 | 0.555 | 0.0441 | No |
| 13 | ZNF668 | zinc finger protein 668 [Source:HGNC Symbol;Acc:HGNC:25821] | 955 | 0.527 | 0.0423 | No |
| 14 | HOXD9 | homeobox D9 [Source:HGNC Symbol;Acc:HGNC:5140] | 999 | 0.517 | 0.0456 | No |
| 15 | SCRT1 | scratch family transcriptional repressor 1 [Source:HGNC Symbol;Acc:HGNC:15950] | 1174 | 0.481 | 0.0381 | No |
| 16 | EN1 | engrailed homeobox 1 [Source:HGNC Symbol;Acc:HGNC:3342] | 1251 | 0.467 | 0.0382 | No |
| 17 | ZNF20 | zinc finger protein 20 [Source:HGNC Symbol;Acc:HGNC:12992] | 1414 | 0.441 | 0.0311 | No |
| 18 | ZBTB1 | zinc finger and BTB domain containing 1 [Source:HGNC Symbol;Acc:HGNC:20259] | 1483 | 0.431 | 0.0313 | No |
| 19 | MLX | MAX dimerization protein MLX [Source:HGNC Symbol;Acc:HGNC:11645] | 1604 | 0.413 | 0.0271 | No |
| 20 | ZNF688 | zinc finger protein 688 [Source:HGNC Symbol;Acc:HGNC:30489] | 1897 | 0.373 | 0.0089 | No |
| 21 | FOXD3 | forkhead box D3 [Source:HGNC Symbol;Acc:HGNC:3804] | 1964 | 0.366 | 0.0084 | No |
| 22 | ZNF85 | zinc finger protein 85 [Source:HGNC Symbol;Acc:HGNC:13160] | 2096 | 0.350 | 0.0026 | No |
| 23 | CTCF | CCCTC-binding factor [Source:HGNC Symbol;Acc:HGNC:13723] | 2183 | 0.340 | 0.0002 | No |
| 24 | ZBTB5 | zinc finger and BTB domain containing 5 [Source:HGNC Symbol;Acc:HGNC:23836] | 2197 | 0.339 | 0.0036 | No |
| 25 | DEAF1 | DEAF1 transcription factor [Source:HGNC Symbol;Acc:HGNC:14677] | 2216 | 0.337 | 0.0065 | No |
| 26 | NR2F6 | nuclear receptor subfamily 2 group F member 6 [Source:HGNC Symbol;Acc:HGNC:7977] | 2238 | 0.335 | 0.0092 | No |
| 27 | FEZF2 | FEZ family zinc finger 2 [Source:HGNC Symbol;Acc:HGNC:13506] | 2278 | 0.329 | 0.0103 | No |
| 28 | ZNF750 | zinc finger protein 750 [Source:HGNC Symbol;Acc:HGNC:25843] | 2449 | 0.308 | 0.0009 | No |
| 29 | INSM1 | INSM transcriptional repressor 1 [Source:HGNC Symbol;Acc:HGNC:6090] | 2531 | 0.299 | -0.0016 | No |
| 30 | ZNF140 | zinc finger protein 140 [Source:HGNC Symbol;Acc:HGNC:12925] | 2609 | 0.290 | -0.0040 | No |
| 31 | NACC2 | NACC family member 2 [Source:HGNC Symbol;Acc:HGNC:23846] | 2655 | 0.284 | -0.0038 | No |
| 32 | DLX4 | distal-less homeobox 4 [Source:HGNC Symbol;Acc:HGNC:2917] | 2660 | 0.284 | -0.0005 | No |
| 33 | ZFP69B | ZFP69 zinc finger protein B [Source:HGNC Symbol;Acc:HGNC:28053] | 2678 | 0.282 | 0.0018 | No |
| 34 | HMX1 | H6 family homeobox 1 [Source:HGNC Symbol;Acc:HGNC:5017] | 2698 | 0.279 | 0.0039 | No |
| 35 | THAP11 | THAP domain containing 11 [Source:HGNC Symbol;Acc:HGNC:23194] | 2800 | 0.270 | -0.0005 | No |
| 36 | ZNF589 | zinc finger protein 589 [Source:HGNC Symbol;Acc:HGNC:16747] | 2873 | 0.263 | -0.0028 | No |
| 37 | VAX2 | ventral anterior homeobox 2 [Source:HGNC Symbol;Acc:HGNC:12661] | 2950 | 0.255 | -0.0055 | No |
| 38 | NFATC3 | nuclear factor of activated T cells 3 [Source:HGNC Symbol;Acc:HGNC:7777] | 3079 | 0.242 | -0.0125 | No |
| 39 | ZNF764 | zinc finger protein 764 [Source:HGNC Symbol;Acc:HGNC:28200] | 3183 | 0.233 | -0.0176 | No |
| 40 | ZNF175 | zinc finger protein 175 [Source:HGNC Symbol;Acc:HGNC:12964] | 3266 | 0.224 | -0.0212 | No |
| 41 | IRF8 | interferon regulatory factor 8 [Source:HGNC Symbol;Acc:HGNC:5358] | 3504 | 0.202 | -0.0373 | No |
| 42 | CUX2 | cut like homeobox 2 [Source:HGNC Symbol;Acc:HGNC:19347] | 3521 | 0.200 | -0.0360 | No |
| 43 | ZNF343 | zinc finger protein 343 [Source:HGNC Symbol;Acc:HGNC:16017] | 3528 | 0.200 | -0.0339 | No |
| 44 | PRRX1 | paired related homeobox 1 [Source:HGNC Symbol;Acc:HGNC:9142] | 3657 | 0.189 | -0.0415 | No |
| 45 | RORC | RAR related orphan receptor C [Source:HGNC Symbol;Acc:HGNC:10260] | 3688 | 0.186 | -0.0415 | No |
| 46 | BCL11A | BAF chromatin remodeling complex subunit BCL11A [Source:HGNC Symbol;Acc:HGNC:13221] | 3781 | 0.179 | -0.0464 | No |
| 47 | MAX | MYC associated factor X [Source:HGNC Symbol;Acc:HGNC:6913] | 3804 | 0.176 | -0.0459 | No |
| 48 | ZBTB14 | zinc finger and BTB domain containing 14 [Source:HGNC Symbol;Acc:HGNC:12860] | 3814 | 0.175 | -0.0443 | No |
| 49 | PEG3 | paternally expressed 3 [Source:HGNC Symbol;Acc:HGNC:8826] | 3823 | 0.174 | -0.0427 | No |
| 50 | ZNF536 | zinc finger protein 536 [Source:HGNC Symbol;Acc:HGNC:29025] | 3839 | 0.172 | -0.0417 | No |
| 51 | ZBTB6 | zinc finger and BTB domain containing 6 [Source:HGNC Symbol;Acc:HGNC:16764] | 3868 | 0.168 | -0.0417 | No |
| 52 | SALL1 | spalt like transcription factor 1 [Source:HGNC Symbol;Acc:HGNC:10524] | 3930 | 0.162 | -0.0445 | No |
| 53 | RELA | "RELA proto-oncogene, NF-kB subunit [Source:HGNC Symbol;Acc:HGNC:9955]" | 3942 | 0.161 | -0.0432 | No |
| 54 | ZNF674 | zinc finger protein 674 [Source:HGNC Symbol;Acc:HGNC:17625] | 3954 | 0.160 | -0.0420 | No |
| 55 | HEY2 | hes related family bHLH transcription factor with YRPW motif 2 [Source:HGNC Symbol;Acc:HGNC:4881] | 4058 | 0.150 | -0.0482 | No |
| 56 | SMAD3 | SMAD family member 3 [Source:HGNC Symbol;Acc:HGNC:6769] | 4061 | 0.150 | -0.0465 | No |
| 57 | MXD1 | MAX dimerization protein 1 [Source:HGNC Symbol;Acc:HGNC:6761] | 4160 | 0.141 | -0.0524 | No |
| 58 | MXI1 | "MAX interactor 1, dimerization protein [Source:HGNC Symbol;Acc:HGNC:7534]" | 4164 | 0.141 | -0.0508 | No |
| 59 | ZNF614 | zinc finger protein 614 [Source:HGNC Symbol;Acc:HGNC:24722] | 4236 | 0.134 | -0.0547 | No |
| 60 | PROX1 | prospero homeobox 1 [Source:HGNC Symbol;Acc:HGNC:9459] | 4299 | 0.128 | -0.0579 | No |
| 61 | TFEC | transcription factor EC [Source:HGNC Symbol;Acc:HGNC:11754] | 4306 | 0.128 | -0.0567 | No |
| 62 | TCF21 | transcription factor 21 [Source:HGNC Symbol;Acc:HGNC:11632] | 4321 | 0.127 | -0.0562 | No |
| 63 | NEUROG3 | neurogenin 3 [Source:HGNC Symbol;Acc:HGNC:13806] | 4443 | 0.118 | -0.0642 | No |
| 64 | NFKB1 | nuclear factor kappa B subunit 1 [Source:HGNC Symbol;Acc:HGNC:7794] | 4626 | 0.102 | -0.0773 | No |
| 65 | ZNF282 | zinc finger protein 282 [Source:HGNC Symbol;Acc:HGNC:13076] | 4725 | 0.093 | -0.0838 | No |
| 66 | MITF | melanocyte inducing transcription factor [Source:HGNC Symbol;Acc:HGNC:7105] | 4759 | 0.090 | -0.0852 | No |
| 67 | TBX3 | T-box transcription factor 3 [Source:HGNC Symbol;Acc:HGNC:11602] | 4790 | 0.088 | -0.0865 | No |
| 68 | HEYL | hes related family bHLH transcription factor with YRPW motif like [Source:HGNC Symbol;Acc:HGNC:4882] | 4820 | 0.086 | -0.0876 | No |
| 69 | NFX1 | "nuclear transcription factor, X-box binding 1 [Source:HGNC Symbol;Acc:HGNC:7803]" | 4930 | 0.078 | -0.0952 | No |
| 70 | BACH2 | BTB domain and CNC homolog 2 [Source:HGNC Symbol;Acc:HGNC:14078] | 4993 | 0.074 | -0.0992 | No |
| 71 | OVOL2 | ovo like zinc finger 2 [Source:HGNC Symbol;Acc:HGNC:15804] | 5017 | 0.071 | -0.1001 | No |
| 72 | ASCL2 | achaete-scute family bHLH transcription factor 2 [Source:HGNC Symbol;Acc:HGNC:739] | 5194 | 0.057 | -0.1132 | No |
| 73 | NFE2L3 | "nuclear factor, erythroid 2 like 3 [Source:HGNC Symbol;Acc:HGNC:7783]" | 5201 | 0.056 | -0.1130 | No |
| 74 | NKX3-2 | NK3 homeobox 2 [Source:HGNC Symbol;Acc:HGNC:951] | 5236 | 0.053 | -0.1150 | No |
| 75 | TFAP2A | transcription factor AP-2 alpha [Source:HGNC Symbol;Acc:HGNC:11742] | 5379 | 0.041 | -0.1256 | No |
| 76 | TP53 | tumor protein p53 [Source:HGNC Symbol;Acc:HGNC:11998] | 5511 | 0.032 | -0.1356 | No |
| 77 | E2F8 | E2F transcription factor 8 [Source:HGNC Symbol;Acc:HGNC:24727] | 5527 | 0.031 | -0.1363 | No |
| 78 | ZNF556 | zinc finger protein 556 [Source:HGNC Symbol;Acc:HGNC:25669] | 5558 | 0.028 | -0.1384 | No |
| 79 | ZBTB10 | zinc finger and BTB domain containing 10 [Source:HGNC Symbol;Acc:HGNC:30953] | 5574 | 0.026 | -0.1392 | No |
| 80 | ZBTB18 | zinc finger and BTB domain containing 18 [Source:HGNC Symbol;Acc:HGNC:13030] | 5782 | 0.009 | -0.1554 | No |
| 81 | ZFP37 | ZFP37 zinc finger protein [Source:HGNC Symbol;Acc:HGNC:12863] | 5804 | 0.008 | -0.1570 | No |
| 82 | ZNF391 | zinc finger protein 391 [Source:HGNC Symbol;Acc:HGNC:18779] | 5813 | 0.008 | -0.1575 | No |
| 83 | ZIM2 | zinc finger imprinted 2 [Source:HGNC Symbol;Acc:HGNC:12875] | 5882 | 0.003 | -0.1628 | No |
| 84 | KLF12 | Kruppel like factor 12 [Source:HGNC Symbol;Acc:HGNC:6346] | 5940 | -0.001 | -0.1673 | No |
| 85 | ZNF350 | zinc finger protein 350 [Source:HGNC Symbol;Acc:HGNC:16656] | 5965 | -0.002 | -0.1692 | No |
| 86 | PPARG | peroxisome proliferator activated receptor gamma [Source:HGNC Symbol;Acc:HGNC:9236] | 6006 | -0.005 | -0.1723 | No |
| 87 | ZNF177 | zinc finger protein 177 [Source:HGNC Symbol;Acc:HGNC:12966] | 6156 | -0.017 | -0.1838 | No |
| 88 | ETV3 | ETS variant transcription factor 3 [Source:HGNC Symbol;Acc:HGNC:3492] | 6206 | -0.021 | -0.1874 | No |
| 89 | ZBTB32 | zinc finger and BTB domain containing 32 [Source:HGNC Symbol;Acc:HGNC:16763] | 6278 | -0.026 | -0.1927 | No |
| 90 | ZNF124 | zinc finger protein 124 [Source:HGNC Symbol;Acc:HGNC:12907] | 6483 | -0.041 | -0.2082 | No |
| 91 | FOXO1 | forkhead box O1 [Source:HGNC Symbol;Acc:HGNC:3819] | 6511 | -0.043 | -0.2098 | No |
| 92 | SMAD5 | SMAD family member 5 [Source:HGNC Symbol;Acc:HGNC:6771] | 6530 | -0.045 | -0.2107 | No |
| 93 | CC2D1A | coiled-coil and C2 domain containing 1A [Source:HGNC Symbol;Acc:HGNC:30237] | 6541 | -0.045 | -0.2109 | No |
| 94 | ZNF133 | zinc finger protein 133 [Source:HGNC Symbol;Acc:HGNC:12917] | 6590 | -0.049 | -0.2140 | No |
| 95 | HINFP | histone H4 transcription factor [Source:HGNC Symbol;Acc:HGNC:17850] | 6776 | -0.061 | -0.2278 | No |
| 96 | MSC | musculin [Source:HGNC Symbol;Acc:HGNC:7321] | 6794 | -0.062 | -0.2284 | No |
| 97 | AEBP1 | AE binding protein 1 [Source:HGNC Symbol;Acc:HGNC:303] | 6822 | -0.065 | -0.2297 | No |
| 98 | E2F6 | E2F transcription factor 6 [Source:HGNC Symbol;Acc:HGNC:3120] | 6832 | -0.065 | -0.2295 | No |
| 99 | BACH1 | BTB domain and CNC homolog 1 [Source:HGNC Symbol;Acc:HGNC:935] | 6909 | -0.073 | -0.2346 | No |
| 100 | HDGF | heparin binding growth factor [Source:HGNC Symbol;Acc:HGNC:4856] | 6924 | -0.074 | -0.2347 | No |
| 101 | ASCL3 | achaete-scute family bHLH transcription factor 3 [Source:HGNC Symbol;Acc:HGNC:740] | 6925 | -0.074 | -0.2338 | No |
| 102 | NR1D1 | nuclear receptor subfamily 1 group D member 1 [Source:HGNC Symbol;Acc:HGNC:7962] | 6974 | -0.078 | -0.2365 | No |
| 103 | ZBTB33 | zinc finger and BTB domain containing 33 [Source:HGNC Symbol;Acc:HGNC:16682] | 7019 | -0.081 | -0.2390 | No |
| 104 | SP3 | Sp3 transcription factor [Source:HGNC Symbol;Acc:HGNC:11208] | 7033 | -0.082 | -0.2389 | No |
| 105 | FOXP3 | forkhead box P3 [Source:HGNC Symbol;Acc:HGNC:6106] | 7035 | -0.082 | -0.2379 | No |
| 106 | MYT1L | myelin transcription factor 1 like [Source:HGNC Symbol;Acc:HGNC:7623] | 7190 | -0.095 | -0.2489 | No |
| 107 | TBX21 | T-box transcription factor 21 [Source:HGNC Symbol;Acc:HGNC:11599] | 7207 | -0.097 | -0.2489 | No |
| 108 | SP2 | Sp2 transcription factor [Source:HGNC Symbol;Acc:HGNC:11207] | 7209 | -0.097 | -0.2477 | No |
| 109 | CREB3L1 | cAMP responsive element binding protein 3 like 1 [Source:HGNC Symbol;Acc:HGNC:18856] | 7324 | -0.104 | -0.2554 | No |
| 110 | SKI | SKI proto-oncogene [Source:HGNC Symbol;Acc:HGNC:10896] | 7349 | -0.106 | -0.2559 | No |
| 111 | ASCL1 | achaete-scute family bHLH transcription factor 1 [Source:HGNC Symbol;Acc:HGNC:738] | 7375 | -0.108 | -0.2565 | No |
| 112 | ZNF180 | zinc finger protein 180 [Source:HGNC Symbol;Acc:HGNC:12970] | 7379 | -0.109 | -0.2553 | No |
| 113 | HIC2 | HIC ZBTB transcriptional repressor 2 [Source:HGNC Symbol;Acc:HGNC:18595] | 7408 | -0.111 | -0.2561 | No |
| 114 | ZNF148 | zinc finger protein 148 [Source:HGNC Symbol;Acc:HGNC:12933] | 7442 | -0.114 | -0.2572 | No |
| 115 | LEF1 | lymphoid enhancer binding factor 1 [Source:HGNC Symbol;Acc:HGNC:6551] | 7467 | -0.116 | -0.2576 | No |
| 116 | TCF3 | transcription factor 3 [Source:HGNC Symbol;Acc:HGNC:11633] | 7521 | -0.120 | -0.2602 | No |
| 117 | SATB1 | SATB homeobox 1 [Source:HGNC Symbol;Acc:HGNC:10541] | 7586 | -0.126 | -0.2636 | No |
| 118 | HES2 | hes family bHLH transcription factor 2 [Source:HGNC Symbol;Acc:HGNC:16005] | 7610 | -0.128 | -0.2638 | No |
| 119 | NR2E1 | nuclear receptor subfamily 2 group E member 1 [Source:HGNC Symbol;Acc:HGNC:7973] | 7660 | -0.132 | -0.2660 | No |
| 120 | GFI1 | growth factor independent 1 transcriptional repressor [Source:HGNC Symbol;Acc:HGNC:4237] | 7901 | -0.147 | -0.2830 | No |
| 121 | ELK3 | ETS transcription factor ELK3 [Source:HGNC Symbol;Acc:HGNC:3325] | 7921 | -0.149 | -0.2826 | No |
| 122 | PLAGL1 | PLAG1 like zinc finger 1 [Source:HGNC Symbol;Acc:HGNC:9046] | 8025 | -0.157 | -0.2887 | No |
| 123 | CREM | cAMP responsive element modulator [Source:HGNC Symbol;Acc:HGNC:2352] | 8026 | -0.157 | -0.2867 | No |
| 124 | ZEB1 | zinc finger E-box binding homeobox 1 [Source:HGNC Symbol;Acc:HGNC:11642] | 8147 | -0.166 | -0.2940 | No |
| 125 | ZNF239 | zinc finger protein 239 [Source:HGNC Symbol;Acc:HGNC:13031] | 8182 | -0.170 | -0.2945 | No |
| 126 | HEY1 | hes related family bHLH transcription factor with YRPW motif 1 [Source:HGNC Symbol;Acc:HGNC:4880] | 8213 | -0.172 | -0.2946 | No |
| 127 | ZNF134 | zinc finger protein 134 [Source:HGNC Symbol;Acc:HGNC:12918] | 8247 | -0.175 | -0.2950 | No |
| 128 | POU4F2 | POU class 4 homeobox 2 [Source:HGNC Symbol;Acc:HGNC:9219] | 8292 | -0.178 | -0.2961 | No |
| 129 | NR2F1 | nuclear receptor subfamily 2 group F member 1 [Source:HGNC Symbol;Acc:HGNC:7975] | 8320 | -0.179 | -0.2959 | No |
| 130 | ZNF205 | zinc finger protein 205 [Source:HGNC Symbol;Acc:HGNC:12996] | 8465 | -0.191 | -0.3048 | No |
| 131 | PRDM5 | PR/SET domain 5 [Source:HGNC Symbol;Acc:HGNC:9349] | 8486 | -0.193 | -0.3039 | No |
| 132 | NANOG | Nanog homeobox [Source:HGNC Symbol;Acc:HGNC:20857] | 8579 | -0.200 | -0.3086 | No |
| 133 | PATZ1 | POZ/BTB and AT hook containing zinc finger 1 [Source:HGNC Symbol;Acc:HGNC:13071] | 8589 | -0.200 | -0.3067 | No |
| 134 | ZBTB39 | zinc finger and BTB domain containing 39 [Source:HGNC Symbol;Acc:HGNC:29014] | 8627 | -0.203 | -0.3070 | No |
| 135 | TRPS1 | transcriptional repressor GATA binding 1 [Source:HGNC Symbol;Acc:HGNC:12340] | 8738 | -0.212 | -0.3130 | No |
| 136 | TFAP2C | transcription factor AP-2 gamma [Source:HGNC Symbol;Acc:HGNC:11744] | 8824 | -0.219 | -0.3169 | No |
| 137 | NR2C1 | nuclear receptor subfamily 2 group C member 1 [Source:HGNC Symbol;Acc:HGNC:7971] | 8940 | -0.227 | -0.3230 | No |
| 138 | PRDM2 | PR/SET domain 2 [Source:HGNC Symbol;Acc:HGNC:9347] | 8992 | -0.231 | -0.3240 | No |
| 139 | SPI1 | Spi-1 proto-oncogene [Source:HGNC Symbol;Acc:HGNC:11241] | 9012 | -0.232 | -0.3225 | No |
| 140 | HAND1 | heart and neural crest derivatives expressed 1 [Source:HGNC Symbol;Acc:HGNC:4807] | 9082 | -0.238 | -0.3249 | No |
| 141 | MXD3 | MAX dimerization protein 3 [Source:HGNC Symbol;Acc:HGNC:14008] | 9122 | -0.241 | -0.3249 | No |
| 142 | POU4F1 | POU class 4 homeobox 1 [Source:HGNC Symbol;Acc:HGNC:9218] | 9300 | -0.256 | -0.3355 | No |
| 143 | ZFHX3 | zinc finger homeobox 3 [Source:HGNC Symbol;Acc:HGNC:777] | 9436 | -0.268 | -0.3427 | No |
| 144 | GTF2IRD1 | GTF2I repeat domain containing 1 [Source:HGNC Symbol;Acc:HGNC:4661] | 9497 | -0.274 | -0.3439 | No |
| 145 | ZBED2 | zinc finger BED-type containing 2 [Source:HGNC Symbol;Acc:HGNC:20710] | 9527 | -0.276 | -0.3426 | No |
| 146 | KLF8 | Kruppel like factor 8 [Source:HGNC Symbol;Acc:HGNC:6351] | 9573 | -0.280 | -0.3426 | No |
| 147 | ENO1 | enolase 1 [Source:HGNC Symbol;Acc:HGNC:3350] | 9606 | -0.284 | -0.3414 | No |
| 148 | SNAI2 | snail family transcriptional repressor 2 [Source:HGNC Symbol;Acc:HGNC:11094] | 9697 | -0.291 | -0.3448 | No |
| 149 | MLXIPL | MLX interacting protein like [Source:HGNC Symbol;Acc:HGNC:12744] | 9882 | -0.310 | -0.3553 | No |
| 150 | NR1D2 | nuclear receptor subfamily 1 group D member 2 [Source:HGNC Symbol;Acc:HGNC:7963] | 9968 | -0.318 | -0.3579 | Yes |
| 151 | ZKSCAN3 | zinc finger with KRAB and SCAN domains 3 [Source:HGNC Symbol;Acc:HGNC:13853] | 9970 | -0.318 | -0.3539 | Yes |
| 152 | SREBF2 | sterol regulatory element binding transcription factor 2 [Source:HGNC Symbol;Acc:HGNC:11290] | 10070 | -0.328 | -0.3575 | Yes |
| 153 | CDX2 | caudal type homeobox 2 [Source:HGNC Symbol;Acc:HGNC:1806] | 10078 | -0.328 | -0.3538 | Yes |
| 154 | IKZF5 | IKAROS family zinc finger 5 [Source:HGNC Symbol;Acc:HGNC:14283] | 10109 | -0.331 | -0.3519 | Yes |
| 155 | RARA | retinoic acid receptor alpha [Source:HGNC Symbol;Acc:HGNC:9864] | 10141 | -0.335 | -0.3500 | Yes |
| 156 | ZNF266 | zinc finger protein 266 [Source:HGNC Symbol;Acc:HGNC:13059] | 10166 | -0.337 | -0.3475 | Yes |
| 157 | ZNF587B | zinc finger protein 587B [Source:HGNC Symbol;Acc:HGNC:37142] | 10198 | -0.340 | -0.3456 | Yes |
| 158 | PRDM16 | PR/SET domain 16 [Source:HGNC Symbol;Acc:HGNC:14000] | 10211 | -0.342 | -0.3421 | Yes |
| 159 | ZBTB16 | zinc finger and BTB domain containing 16 [Source:HGNC Symbol;Acc:HGNC:12930] | 10246 | -0.346 | -0.3403 | Yes |
| 160 | ZNF749 | zinc finger protein 749 [Source:HGNC Symbol;Acc:HGNC:32783] | 10325 | -0.353 | -0.3419 | Yes |
| 161 | PRDM1 | PR/SET domain 1 [Source:HGNC Symbol;Acc:HGNC:9346] | 10374 | -0.358 | -0.3411 | Yes |
| 162 | ZNF224 | zinc finger protein 224 [Source:HGNC Symbol;Acc:HGNC:13017] | 10484 | -0.371 | -0.3449 | Yes |
| 163 | ZNF263 | zinc finger protein 263 [Source:HGNC Symbol;Acc:HGNC:13056] | 10623 | -0.385 | -0.3508 | Yes |
| 164 | OVOL1 | ovo like transcriptional repressor 1 [Source:HGNC Symbol;Acc:HGNC:8525] | 10644 | -0.387 | -0.3474 | Yes |
| 165 | ZNF219 | zinc finger protein 219 [Source:HGNC Symbol;Acc:HGNC:13011] | 10804 | -0.406 | -0.3547 | Yes |
| 166 | ZNF337 | zinc finger protein 337 [Source:HGNC Symbol;Acc:HGNC:15809] | 10810 | -0.406 | -0.3498 | Yes |
| 167 | IRF3 | interferon regulatory factor 3 [Source:HGNC Symbol;Acc:HGNC:6118] | 10879 | -0.414 | -0.3498 | Yes |
| 168 | POU5F1 | POU class 5 homeobox 1 [Source:HGNC Symbol;Acc:HGNC:9221] | 10898 | -0.416 | -0.3459 | Yes |
| 169 | MNT | MAX network transcriptional repressor [Source:HGNC Symbol;Acc:HGNC:7188] | 10952 | -0.424 | -0.3446 | Yes |
| 170 | TGIF1 | TGFB induced factor homeobox 1 [Source:HGNC Symbol;Acc:HGNC:11776] | 10957 | -0.425 | -0.3394 | Yes |
| 171 | POU6F1 | POU class 6 homeobox 1 [Source:HGNC Symbol;Acc:HGNC:9224] | 10989 | -0.428 | -0.3363 | Yes |
| 172 | ZBTB17 | zinc finger and BTB domain containing 17 [Source:HGNC Symbol;Acc:HGNC:12936] | 10996 | -0.429 | -0.3313 | Yes |
| 173 | ETS2 | "ETS proto-oncogene 2, transcription factor [Source:HGNC Symbol;Acc:HGNC:3489]" | 11057 | -0.439 | -0.3303 | Yes |
| 174 | PPARA | peroxisome proliferator activated receptor alpha [Source:HGNC Symbol;Acc:HGNC:9232] | 11110 | -0.445 | -0.3287 | Yes |
| 175 | HOXA2 | homeobox A2 [Source:HGNC Symbol;Acc:HGNC:5103] | 11183 | -0.455 | -0.3285 | Yes |
| 176 | HSF1 | heat shock transcription factor 1 [Source:HGNC Symbol;Acc:HGNC:5224] | 11189 | -0.456 | -0.3230 | Yes |
| 177 | ZNF550 | zinc finger protein 550 [Source:HGNC Symbol;Acc:HGNC:28643] | 11224 | -0.460 | -0.3197 | Yes |
| 178 | SKIL | SKI like proto-oncogene [Source:HGNC Symbol;Acc:HGNC:10897] | 11253 | -0.464 | -0.3160 | Yes |
| 179 | ZNF217 | zinc finger protein 217 [Source:HGNC Symbol;Acc:HGNC:13009] | 11265 | -0.466 | -0.3108 | Yes |
| 180 | PPARD | peroxisome proliferator activated receptor delta [Source:HGNC Symbol;Acc:HGNC:9235] | 11301 | -0.471 | -0.3075 | Yes |
| 181 | FOXO3 | forkhead box O3 [Source:HGNC Symbol;Acc:HGNC:3821] | 11305 | -0.472 | -0.3016 | Yes |
| 182 | NKX6-1 | NK6 homeobox 1 [Source:HGNC Symbol;Acc:HGNC:7839] | 11314 | -0.473 | -0.2962 | Yes |
| 183 | BATF3 | basic leucine zipper ATF-like transcription factor 3 [Source:HGNC Symbol;Acc:HGNC:28915] | 11353 | -0.479 | -0.2930 | Yes |
| 184 | NFATC4 | nuclear factor of activated T cells 4 [Source:HGNC Symbol;Acc:HGNC:7778] | 11363 | -0.481 | -0.2875 | Yes |
| 185 | PROP1 | PROP paired-like homeobox 1 [Source:HGNC Symbol;Acc:HGNC:9455] | 11373 | -0.482 | -0.2819 | Yes |
| 186 | ZNF778 | zinc finger protein 778 [Source:HGNC Symbol;Acc:HGNC:26479] | 11436 | -0.493 | -0.2805 | Yes |
| 187 | ZNF248 | zinc finger protein 248 [Source:HGNC Symbol;Acc:HGNC:13041] | 11500 | -0.501 | -0.2790 | Yes |
| 188 | ZBTB7A | zinc finger and BTB domain containing 7A [Source:HGNC Symbol;Acc:HGNC:18078] | 11600 | -0.516 | -0.2801 | Yes |
| 189 | FOXK2 | forkhead box K2 [Source:HGNC Symbol;Acc:HGNC:6036] | 11609 | -0.517 | -0.2741 | Yes |
| 190 | ZEB2 | zinc finger E-box binding homeobox 2 [Source:HGNC Symbol;Acc:HGNC:14881] | 11644 | -0.524 | -0.2700 | Yes |
| 191 | TBX2 | T-box transcription factor 2 [Source:HGNC Symbol;Acc:HGNC:11597] | 11728 | -0.538 | -0.2696 | Yes |
| 192 | HOXB13 | homeobox B13 [Source:HGNC Symbol;Acc:HGNC:5112] | 11766 | -0.544 | -0.2655 | Yes |
| 193 | DACH1 | dachshund family transcription factor 1 [Source:HGNC Symbol;Acc:HGNC:2663] | 11843 | -0.558 | -0.2643 | Yes |
| 194 | ETV7 | ETS variant transcription factor 7 [Source:HGNC Symbol;Acc:HGNC:18160] | 11872 | -0.566 | -0.2592 | Yes |
| 195 | ZBTB20 | zinc finger and BTB domain containing 20 [Source:HGNC Symbol;Acc:HGNC:13503] | 11902 | -0.572 | -0.2541 | Yes |
| 196 | HIVEP1 | HIVEP zinc finger 1 [Source:HGNC Symbol;Acc:HGNC:4920] | 11911 | -0.574 | -0.2473 | Yes |
| 197 | ZNF202 | zinc finger protein 202 [Source:HGNC Symbol;Acc:HGNC:12994] | 11946 | -0.582 | -0.2425 | Yes |
| 198 | ZNF3 | zinc finger protein 3 [Source:HGNC Symbol;Acc:HGNC:13089] | 11990 | -0.591 | -0.2382 | Yes |
| 199 | ERF | ETS2 repressor factor [Source:HGNC Symbol;Acc:HGNC:3444] | 12044 | -0.605 | -0.2346 | Yes |
| 200 | MSX1 | msh homeobox 1 [Source:HGNC Symbol;Acc:HGNC:7391] | 12054 | -0.609 | -0.2275 | Yes |
| 201 | HIC1 | HIC ZBTB transcriptional repressor 1 [Source:HGNC Symbol;Acc:HGNC:4909] | 12118 | -0.624 | -0.2244 | Yes |
| 202 | ZBTB25 | zinc finger and BTB domain containing 25 [Source:HGNC Symbol;Acc:HGNC:13112] | 12168 | -0.635 | -0.2200 | Yes |
| 203 | YY1 | YY1 transcription factor [Source:HGNC Symbol;Acc:HGNC:12856] | 12229 | -0.653 | -0.2163 | Yes |
| 204 | ETV6 | ETS variant transcription factor 6 [Source:HGNC Symbol;Acc:HGNC:3495] | 12231 | -0.654 | -0.2080 | Yes |
| 205 | ZNF79 | zinc finger protein 79 [Source:HGNC Symbol;Acc:HGNC:13153] | 12234 | -0.655 | -0.1997 | Yes |
| 206 | GATA3 | GATA binding protein 3 [Source:HGNC Symbol;Acc:HGNC:4172] | 12276 | -0.670 | -0.1942 | Yes |
| 207 | SOX13 | SRY-box transcription factor 13 [Source:HGNC Symbol;Acc:HGNC:11192] | 12338 | -0.688 | -0.1902 | Yes |
| 208 | SNAI1 | snail family transcriptional repressor 1 [Source:HGNC Symbol;Acc:HGNC:11128] | 12365 | -0.696 | -0.1832 | Yes |
| 209 | REST | RE1 silencing transcription factor [Source:HGNC Symbol;Acc:HGNC:9966] | 12382 | -0.703 | -0.1754 | Yes |
| 210 | ZNF440 | zinc finger protein 440 [Source:HGNC Symbol;Acc:HGNC:20874] | 12499 | -0.748 | -0.1749 | Yes |
| 211 | MZF1 | myeloid zinc finger 1 [Source:HGNC Symbol;Acc:HGNC:13108] | 12528 | -0.762 | -0.1673 | Yes |
| 212 | BHLHE40 | basic helix-loop-helix family member e40 [Source:HGNC Symbol;Acc:HGNC:1046] | 12573 | -0.782 | -0.1607 | Yes |
| 213 | ZNF131 | zinc finger protein 131 [Source:HGNC Symbol;Acc:HGNC:12915] | 12585 | -0.786 | -0.1514 | Yes |
| 214 | ZNF692 | zinc finger protein 692 [Source:HGNC Symbol;Acc:HGNC:26049] | 12617 | -0.808 | -0.1434 | Yes |
| 215 | ZNF512B | zinc finger protein 512B [Source:HGNC Symbol;Acc:HGNC:29212] | 12623 | -0.809 | -0.1333 | Yes |
| 216 | TCF7 | transcription factor 7 [Source:HGNC Symbol;Acc:HGNC:11639] | 12628 | -0.813 | -0.1231 | Yes |
| 217 | ATF3 | activating transcription factor 3 [Source:HGNC Symbol;Acc:HGNC:785] | 12659 | -0.833 | -0.1147 | Yes |
| 218 | BCL6 | BCL6 transcription repressor [Source:HGNC Symbol;Acc:HGNC:1001] | 12667 | -0.838 | -0.1045 | Yes |
| 219 | HES1 | hes family bHLH transcription factor 1 [Source:HGNC Symbol;Acc:HGNC:5192] | 12676 | -0.849 | -0.0941 | Yes |
| 220 | ZNF10 | zinc finger protein 10 [Source:HGNC Symbol;Acc:HGNC:12879] | 12684 | -0.853 | -0.0837 | Yes |
| 221 | LRRFIP1 | LRR binding FLII interacting protein 1 [Source:HGNC Symbol;Acc:HGNC:6702] | 12717 | -0.880 | -0.0748 | Yes |
| 222 | MAFK | MAF bZIP transcription factor K [Source:HGNC Symbol;Acc:HGNC:6782] | 12724 | -0.886 | -0.0639 | Yes |
| 223 | ZNF281 | zinc finger protein 281 [Source:HGNC Symbol;Acc:HGNC:13075] | 12767 | -0.928 | -0.0552 | Yes |
| 224 | MYC | "MYC proto-oncogene, bHLH transcription factor [Source:HGNC Symbol;Acc:HGNC:7553]" | 12801 | -0.970 | -0.0453 | Yes |
| 225 | CEBPB | CCAAT enhancer binding protein beta [Source:HGNC Symbol;Acc:HGNC:1834] | 12804 | -0.973 | -0.0329 | Yes |
| 226 | JUN | "Jun proto-oncogene, AP-1 transcription factor subunit [Source:HGNC Symbol;Acc:HGNC:6204]" | 12813 | -0.994 | -0.0207 | Yes |
| 227 | NFIL3 | "nuclear factor, interleukin 3 regulated [Source:HGNC Symbol;Acc:HGNC:7787]" | 12845 | -1.048 | -0.0096 | Yes |
| 228 | ZNF669 | zinc finger protein 669 [Source:HGNC Symbol;Acc:HGNC:25736] | 12855 | -1.071 | 0.0036 | Yes |
Table: GSEA details [plain text format]

  

Fig 2: GOMF\_DNA\_BINDING\_TRANSCRIPTION\_REPRESSOR\_ACTIVITY      
 Blue-Pink O' Gram in the Space of the Analyzed GeneSet

  

Fig 3: GOMF\_DNA\_BINDING\_TRANSCRIPTION\_REPRESSOR\_ACTIVITY: Random ES distribution      
 Gene set null distribution of ES for **GOMF\_DNA\_BINDING\_TRANSCRIPTION\_REPRESSOR\_ACTIVITY**
